# Melatonin and Other Tryptophan Metabolites Produced by Yeasts: Implications in Cardiovascular and Neurodegenerative Diseases

**DOI:** 10.3389/fmicb.2015.01565

**Published:** 2016-01-19

**Authors:** Ruth Hornedo-Ortega, Ana B. Cerezo, Ana M. Troncoso, M. Carmen Garcia-Parrilla, Albert Mas

**Affiliations:** ^1^Facultad de Farmacia, Universidad de SevillaSevilla, Spain; ^2^Facultad de Enología, Universitat Rovira i VirgiliTarragona, Spain

**Keywords:** wine, beer, VEGF, β-amyloid, α-synuclein, tryptophol, serotonin

## Abstract

Yeast metabolism produces compounds derived from tryptophan, which are found in fermented beverages, such as wine and beer. In particular, melatonin and serotonin, may be relevant due to their bioactivity in humans. Indeed, the former is a neurohormone related to circadian rhythms, which also has a putative protective effect against degenerative diseases. Moreover, serotonin is a neurotransmitter itself, in addition to being a precursor of melatonin synthesis. This paper summarizes data reported on fermented beverages, to evaluate dietary intake. Additionally, the article reviews observed effects of yeast amino acid metabolites on the prevention of neurodegenerative diseases (Alzheimer’s and Parkinson’s) and angiogenesis, focusing on evidence of the molecular mechanism involved and identification of molecular targets.

## Origin, Occurrence, and Dietary Intake

The presence of bioactive compounds in fermented beverages has long been observed and they have been studied with great interest. A large body of research has focused on polyphenols, in particular, since these bioactive compounds are already present in plants and released into fermented products. Yeast also transforms certain other molecules into biologically active compounds. Among these, the case of amino acid tryptophan is of interest, since it is the precursor of at least three biologically active compounds: melatonin, serotonin, and tryptophol (Mas et al., 2014).

Tryptophol is an alcohol produced by the Ehrlich pathway and it has long been detected in appreciable concentrations in wines and beers in the mg/L range (Bartolomé et al., 2000; Monagas et al., 2007). Therefore, its occurrence in beverages is widely recognized. Moreover, tryptophol has also been indicated as a quorum sensing molecule for yeast (Sprague and Winans, 2006).

Just a few years ago, melatonin was detected in wines in much lower levels: within the ng/L range. Not only was it evidenced in wines, but also in other fermented foods, as summarized in **Table 1**. Furthermore, Rodriguez-Naranjo et al. (2011) highlighted that melatonin was produced after alcoholic fermentation, pinpointing the role *Saccharomyces* plays. Indeed, different strains synthetized melatonin at different levels (Rodriguez-Naranjo et al., 2012).

**Table 1:** Concentration of melatonin and other tryptophan metabolites in fermented products.

Compound		Concentration		Reference
	**Food**			
Melatonin	Probiotic yogurt	126.7 ± 9.00	pg/g or pg/mL	Kocadağli et al. (2014)
	Kefir (fermented milk drink)	n.d	pg/g or pg/mL	
	Black olive (naturally fermented)	5.3 ± 0.10	pg/g or pg/mL	
	Bread (crumb)	341.7 ± 29.30	pg/g or pg/mL	
	Bread (crust)	138.1 ± 23.20	pg/g or pg/mL	
	Beer	94.5 ± 6.70	pg/g or pg/mL	
	**Wine**			
	Alaban, Sangiovese, Trebbiano (Italy)	0.6-0.4	ng/mL	Mercolini et al., 2012
	Chardonnay, Malbec, Cabernet Sauvignon (Argentina)	0.16-0.32		Stege et al., 2010
	Gropello, Merlot (Italy)	8.1-5.2		Vitalini et al., 2013
	Cabernet Saivignon, Merlot, Syrah, Tempranillo, Tintilla de Rota, Petit Verdot, Prieto Picudo, and Palomino fino (Spain)	5.1-420		Rodriguez-Naranjo et al., 2011
	Fermented orange beverage	20.0 ± 2.02	ng/mL	Fernández-Pachón et al., 2014
	Red wine	4.88-9.15	mg/L	Monagas et al., 2007
	Fermented lentils	2.70+0.25	mg/g dry material	Bartolomé et al., 2000
Tryptophol	Whole-wheat bread	-		Jiang and Peterson, 2013
	Beer	0.242 ± 0.200	mg/L	Bartolomé et al., 2000
Serotonin	Beer	3.5-24.2	mg/L	Kirschbaum et al., 1999
	Wine	2.94-5.93	mg/L	Wang et al., 2014
		1.93 ± 0.043	mg/L	Manfroi et al., 2009
		5.5	ng/mL	Mandrioli et al., 2011

The synthetic pathway of melatonin in yeast is not completely elucidated, yet it seems the formation of serotonin might be an intermediate in the pathway (Mas et al., 2014). In addition, serotonin has been detected at mg/L levels in red wine following malolactic fermentation (Wang et al., 2014). Further research is required to explore the roles of yeast and bacteria in the occurrence of these bioactive compounds in fermented products.

One of the characteristics of bioactive compounds is the minimal concentration required for them to act. The reported concentrations in wine and beer would mean that someone consuming these beverages would obtain a low daily intake of these compounds. According to WHO, the daily intake of ethanol should not exceed 30 g and 20 g for men and women, respectively. That is to say that daily intake for a man of a wine can provide up to 0.00005–0.13 mg of melatonin. In a comprehensive review summarizing the results of human intervention studies, Harpsøe et al. (2015) concluded that the bioavailability of melatonin was 15%. In our example, its bioavailable concentration should result in 1.5–4000 pg/mL of melatonin in blood. Physiological values for day plasma melatonin are very low, accounting for several pg/mL (5–10 pg/mL in human plasma) (de Almeida et al., 2011). Thus, pg/mL in plasma might be expected after dietetic intake of wine or beer, considering the values displayed in **Table 1**. Indeed, Maldonado et al. (2009) determined an increase in the concentration of plasmatic melatonin after the ingestion of a moderate dose of beer (330 mL for women volunteers, 660 mL for men). To the best of our knowledge, there is no published data on the bioavailability of serotonin after food or beverage intake.

## Biological Effects and Prevention of Chronic Diseases

Literature on the biological effects of these compounds is extensive and encompasses circadian rhythm, antioxidant properties, and reproductive function. Due to the length of this mini-review, we will focus on more recent findings on the prevention of the most prevalent degenerative diseases, such as cancer, and cardiovascular and neurodegenerative diseases.

## Implications for Cancer and Cardiovascular Disease: The Role of Angiogenesis

Angiogenesis, which consists of the formation of new blood vessels from pre-existing ones, is crucial for organ growth during embryonic development and after birth. However, in adulthood, angiogenesis plays an essential role in the pathogenesis of diverse chronic diseases, such as cancer and cardiovascular disease, involving the progression and development of the tumor, and development and destabilization of atherosclerotic plaques (Celletti et al., 2001; Bergers and Benjamin, 2003).

Angiogenesis occurs when there is an imbalance between pro-angiogenic (e.g., vascular endothelial growth factor (VEGF), basic fibroblast growth factor, alfa tumor necrotic factor, etc.) and anti-angiogenic (e.g., angiostatin and endostatin) factors. VEGF is the most active endogenous pro-angiogenic factor in humans (Giles, 2001; Dulak, 2005; Cebe-Suarez et al., 2006; Cook and Figg, 2010). It exerts its angiogenic effect by stimulating VEGF receptor 2 (VEGFR-2), which is critical for promoting the proliferation and differentiation of endothelial cells (Giles, 2001; Ferrara and Kerbel, 2005). It has been demonstrated that VEGF promotes atherosclerotic plaque progression (Celletti et al., 2001; Khurana et al., 2005) and tumor angiogenesis (Senger et al., 1993). Indeed, VEGF is a target for drug therapies that aim to inhibit VEGF signaling (Ferrara and Kerbel, 2005). Anti-VEGF antibodies, aptamers and small molecule VEGFR tyrosine kinase inhibitors have been developed and given regulatory approval for the treatment of colon, lung, breast, kidney, and liver cancer, in addition to neovascular age-related macular degeneration (Giles, 2001; Ferrara and Kerbel, 2005). However, serious side effects, such as hypertension, have been reported with prolonged use of anti-VEGF therapies (Zhu et al., 2007; Wu et al., 2008; Kappers et al., 2010). The use of natural products in reducing VEGF-induced angiogenesis may prove to be more beneficial than the current anti-VEGF drugs available (Moyle et al., 2015).

Melatonin has been associated with a decline in VEGF secretion levels in the serum of advanced cancer patients (Lissoni et al., 2001), in addition to markedly reducing the expression of VEGF in HUVEC and culture cancer cells at 1 μM and 1 mM (Dai et al., 2008; Cui et al., 2012; Álvarez-García et al., 2013; Gonçalves et al., 2014). Melatonin has also been proven to reduce endothelial cell proliferation, invasion, migration, and tube formation, through downregulation of VEGF at 1 mM (Álvarez-García et al., 2013). The possible cell signaling pathway when melatonin inhibits HUVEC proliferation has been related to the following pathway: melatonin receptors/ERK/PI3K/Akt/PKC/NF-kB (Cui et al., 2008). Additionally, Sohn et al. (2015) have recently demonstrated that melatonin (1 mM) upregulates miRNA3195 and miRNA374b, whose overexpression synergistically reduced VEGF production in hypoxic PC-3 prostate cancer cells, indicating the important role of miRNA3195 and miRNA374b in melatonin induced antiangiogenic activity. Melatonin (40 mg/kg) has also shown an antitumor effect on mammary tumor growth in mice after 21 days of treatment; the mice displayed significantly smaller tumor volume and tumor regression (Jardim-Perassi et al., 2014). Additionally, in the same study, a lower expression of VEGFR2 was observed in the melatonin-treated tumors compared to the vehicle-treated tumors. More research is consequently needed to focus on determining the molecular mechanism by which melatonin exerts its angiogenic effect and the molecular target involved.

## Neurodegenerative Diseases

Alzheimer’s disease (AD) and Parkinson’s disease (PD) are the most common human neurodegenerative diseases. In both cases, their incidence increases with age. The aggregation of proteins that results in different fibrillar structures is responsible for these disorders. Specifically, they are owing to the abnormal pathological assembly of amyloid- beta (Aβ), tau and α- synuclein (αS).

Indeed, several studies have demonstrated that protofibrils and oligomers of αS and Aβ are more neurotoxic than fibrils (Pike et al., 1993; Lashuel et al., 2002; Volles and Lansbury, 2003; Outeiro et al., 2008).

This review focus on the evidence of certain bioactives which can present in fermented products. However, to give a fair balance, alcohol effects on neurodegeneration have to be highlighted as it is a major component formed by yeast in alcoholic beverages. It is well-Known that alcohol intake crosses the BBB (Blood–Brain Barrier) easily producing the excessive release of neurotransmitters, oxidative stress and inflammatory response which turns out in neurotoxicity and finally cell death. (Persidsky and Potula, 2014).

## Alzheimer’s Disease

Alzheimer’s disease is a progressive and irreversible neurodegenerative disorder characterized by loss of memory and cognition, abstract thinking, and personality alteration. The etiology of AD is unknown in more than 90% of cases. In the pathogenesis of AD there are two principal hallmarks: neurofibrillary tangles (NFTs) and amyloid plaques. NFT are formed by the intracellular accumulation of phosphorylated tau protein and amyloid plaques, by extracellular accumulation of amyloid β peptides (Hardy and Selkoe, 2002). The amyloid beta peptide is formed via cleavage of the amyloid precursor protein (APP). In the non-amyloidogenic pathway (normal state), APP is cleaved by α-secretase, to generate sAPP (soluble N- terminal fragment), which is neuroprotective as it is involved in the enhancement of synaptogenesis, neurite outgrowth, and neuronal survival. Conversely, in the disease state, APP is cleaved by β and γ secretase, resulting in insoluble beta amyloid peptide, which has high potential for assembly and formation of toxic aggregates (Gandy, 2005).

Several mechanisms have been proposed to explain βA neurotoxicity, such as oxidative stress and loss of endogenous antioxidants (Behl et al., 1994; Abramov and Duchen, 2005; Hamel et al., 2008); mitochondrial damage, depolarization, and mitochondrial permeability transition pore opening (Moreira et al., 2001, 2010; Abramov et al., 2004, 2007); destabilization of intracellular calcium homeostasis in neurons (Bezprozvanny and Mattson, 2008); glial cells (Abramov et al., 2003, 2004), and neuroinflammation (McNaull et al., 2010).

Levels of melatonin and its precursors (serotonin and tryptophan) are significantly decreased in elderly AD individuals and are associated with the emergence of AD (Zhou et al., 2003; Greilberger et al., 2010). A growing body of evidence supports the protective role of melatonin in several molecular mechanisms implicated in the development of AD.

Among these mechanisms, the most significant one is that melatonin prevents amyloid aggregation and overproduction. This neurohormone has a great affinity for Aβ peptide, preventing amyloid fibril formation (Masilamoni et al., 2008), as determined by circular dichroism (CD) spectroscopy, electron microscopy, nuclear magnetic resonance (NMR) and electrospray ionization-mass spectrometry (ESI-MS). In particular, a hydrophobic interaction has been observed between melatonin and Aβ, specifically on the 29–40 residues of the Aβ segment (Skribanek et al., 2001). Additionally, melatonin has inhibitory effects on the formation of secondary β-sheet structures through the disruption of the histidine (His^+^) and aspartate (Asp^-^) salt bridges in Aβ peptide that promote fibril dissolution (Fraser et al., 1991; Huang et al., 1997; Pappolla et al., 1998).

Melatonin presents a great capacity to regulate the synthesis and maturation of APP at different levels by: decreasing its mRNA encoding β-APP (Song and Lahiri, 1997; Lahiri, 1999); blocking cAMP production, which is involved in activating the APP gene promoter, (Husson et al., 2002); and inactivating GSK-3, which promotes α-secretase mediated cleavage of APP, favoring the non-amyloidogenic pathway (McArthur et al., 1997; Zhu et al., 2001; Hoppe et al., 2010). *In vivo* studies with transgenic mice over-expressing APP (in 9–10 months they develop senile plaques) and fed with 0.5 mg/mL of melatonin in their drinking water (3 mL/day) found a reduction in important markers of the disease, including Aβ levels in the brain, and that some animals survived (Matsubara et al., 2003). The amount given to rodents are within the pharmacological dose and out of the range of the dose that can be achieved with moderate consumption of wine. Therefore it cannot be concluded that these effect will be observed in humans after wine intake. Further research is required to obtain the evidence at dietary doses.

Furthermore, melatonin exhibits a protective effect on the cholinergic system. In AD patients, a dramatic decrease of acetylcholine has been observed (Francis et al., 1999), which was related to a decrease in enzyme choline acetyltransferase (ChAT) activity and an increase in acetylcholinesterase (AChE) activity (Bieschke et al., 2005). Indeed, AChE inhibitors increase the synaptic levels of acetylcholine, which is why they are used as a treatment for mild to moderate AD. *In vivo* administration of melatonin in rats (50 mg/kg body weight) has led to significantly reduced AChE activity, with maintenance of calcium levels under conditions of oxidative stress (Masilamoni et al., 2008). Regarding ChAT, melatonin increased its activity, after 4 months of melatonin administration in rats (Feng et al., 2004).

Finally, melatonin reduces Aβ-induced oxidative stress related to reactive oxygen species (ROS) and proinflamatory cytokines, such as IL6 and IL1-β in *in vivo* studies (Masilamoni et al., 2008). As a result of these effects, melatonin protects brain neurons from damage and death by increasing viability in hippocampal neurons and glial cells following treatment with Aβ1–40, Aβ25–40, and Aβ1–28. Moreover, melatonin prevents the death of murine N2a neuroblastoma and PC12 cells by using Aβ25–35 (Pappolla et al., 1997; Ionov et al., 2011).

There is scarce literature available in relation to the activity of other tryptophan metabolites, with indole 3-acetic acid and tryptophol being the only bioactive molecules reported so far. Morshedi et al. (2007) proved the inhibitory effect of these indole derivatives on the amyloid fibrillation of hen egg-white lysozyme, which is another model for exploring the amyloidogenic mechanism.

## Parkinson’s Disease

Parkinson’s disease is the second most common neurodegenerative disorder. Its diagnosis is based on motor abnormalities, such as resting tremor, bradykinesia, and rigidity (Duvoisin, 1992). Indeed, patients present other non-motor symptoms, such as depression, anxiety, and sleep disorders (Jenner et al., 2013). Only 10% of patients have a genetic basis, with 90% being considered sporadic cases. PD is characterized by the degeneration of the subcortical structure of the brain. Specifically, there are significant losses of dopaminergic neurons in the substantia nigra pars compacta (SNpc; Forno, 1996), although other cell populations are also susceptible to the neurodegeneration process.

α-Synuclein (αS) is a 140 amino acid and a highly abundant neuronal protein. It is found as a soluble cytoplasmatic protein associated with synaptic vesicles (Iwai et al., 1995). It is thought that it plays a role in neurotransmission and cognitive function. Although its physiological function is uncertain, the pathology is associated with the accumulation of αS aggregates, which are the main component of Lewy bodies (LBs; Spillantini et al., 1997). LBs are spherical inclusions formed by αS aggregate (99%) and other proteins.

Despite the main risk factor being aging, other possible risk factors include mutation in the SNCA (alpha-synuclein gene) and exposure to environmental toxins. The latter are also linked to metabolic abnormalities involving neurotransmitter systems (dopamine, serotonin, GABA, and glutamate), fatty acids, such as arachidonic acid-cascade, oxidative stress and mitochondrial function (Henchcliffe and Beal, 2008; Quinones and Kaddurah-Daouk, 2009; Kaidanovich-Beilin et al., 2012; Lei and Powers, 2013).

Furthermore, several studies suggest that αS oligomers and protofibrils are an important factor in neurotoxicity in PD. αS protofibrils cause membrane permeabilization, which alters cellular homeostasis and may activate an apoptotic process (Volles and Lansbury, 2003). Indeed, there is evidence to support the capacity of αS to inhibit proteosomal activity, which would prevent elimination of misfolded proteins (Giasson and Lee, 2003).

Substantial evidence also suggests that a significant factor in dopaminergic neuronal loss in the PD brain are ROS, which result from dopamine metabolism, low glutathione concentration and high levels of iron and calcium in the SNpc (Jenner and Olanow, 2006). Additionally, the brain contains high concentrations of polyunsaturated fatty acids, which, under oxidative stress, result in lipid peroxidation and generation of toxic products (Liu et al., 2008).

No treatment is currently available for the prevention or cure of PD. However, a combination of L-DOPA and antioxidants has been recommended to reduce the rate of progression of the disease, due to the decrease in dopamine levels and significant increase of oxidative stress commonly concomitant to this type of disorder (Zhu et al., 2004).

Concerning the role of melatonin in PD, several works have reported different mechanisms of action. Lin et al. (2007) demonstrated that melatonin attenuates arsenite-induced apoptosis by reducing aggregated αS levels in rat brains, by means of Western blot analysis. Additionally, Ishido (2007) showed that melatonin inhibits αS assembly, using immunostaining in rat pheochromocytoma cells.

It is also important to highlight that melatonin dose-dependently inhibits all steps of the αS assembly process. Ono et al. (2012) observed a reduction in the number of fibrils and the corresponding increase of the number of short fibrils and amorphous aggregates (25–250 μM) using electron microscopy and thioflavin S experiments. Indeed, melatonin presents a significant destabilization effect (also dose-dependently), suggesting a decrease in beta-sheet levels. In the same study, the authors performed experiments with primary cultures of mesencephalon and neostriatum with MTT: (3-(4,5-Dimethylthiazol-2-yl)-2,5-Diphenyltetrazolium Bromide) a colorimetric assay for assessing cell metabolic activity. The results showed that melatonin reduced the toxic effects of αS after pretreatment (2–6 days) with an increase in cell viability of between 56 and 97%.

In addition to this, it is well known that melatonin exhibits antioxidant properties (Reiter et al., 1997; Kotler et al., 1998). Cellular injury cause by αS-mediated perturbation of cellular redox reactions is an important mechanism proposed for PD (George et al., 2009). Melatonin has been suggested as a potential therapeutic agent in diseases where oxidative stress is thought to be a major pathogenic factor. Mayo et al. (1998) observed that this hormone was an effective free radical scavenger and that it prevented apoptosis in neuronal cells. Moreover, *in vitro* studies on MPTD-induced (1-methyl-4-phenyl-1,2,3,6-tetrahydropyridine) PD in mice have shown that melatonin protects against excitotoxicity by reducing the autoxidation of dopamine. The administration of melatonin leads to normalization of complex I activity and oxidative status in mitochondria (Escames et al., 2010).

In conclusion, and based on the preceding evidence, we should consider that melatonin presents strong inhibitory effects on protofibril formation and peptide oligomerization.

## Author Contributions

RH-O and AC Literature search and first draft. AT, MG-P, and AM thorough revision and discussion and final document.

## Conflict of Interest Statement

The authors declare that the research was conducted in the absence of any commercial or financial relationships that could be construed as a potential conflict of interest.
